# Heavy metal contamination of selected mining fields in North-Central Nigeria

**DOI:** 10.1016/j.mex.2023.102201

**Published:** 2023-04-26

**Authors:** Muyiwa Michael Orosun, Samuel Oluwagbenga Inuyomi, Mojisola Rachael Usikalu, Hussein Kehinde Okoro, Hitler Louis, Maxwell Omeje, Emmanuel Olusegun Ehinlafa, Kayode John Oyewumi

**Affiliations:** a Department of Physics, University of Ilorin, Ilorin, Kwara State, Nigeria; b Department of Physics and Engineering Physics, Obafemi Awolowo University, Ile-ife, Nigeria; c Department of Physics, Covenant University, Ogun State, Nigeria; d Department of Industrial Chemistry, University of Ilorin, Ilorin, Nigeria; e Department of Chemistry, University of Calabar, Calabar, Nigeria

**Keywords:** Dataset, Potentially toxic element, Soil, Beryllium, Gold, Mining, Human Health Risk Assessment of Heavy Metals

## Abstract

This study evaluates the causes, concentration and the associated health risks of selected heavy metals (HMs) in soil samples collected from beryllium and gold mining fields in Nigeria. The samples of soil were collected manually and analysed by means of Atomic Absorption Spectrophotometry (AAS). Seventy-two (72) samples were analysed which presented varying degrees of concentration of the selected HMs. The analysed HMs are Chromium (Cr), Arsenic (As), Iron (Fe), Cadmium (Cd), Nickel (Ni), Manganese (Mn), Magnesium (Mg), Zinc (Zn), Copper (Cu) and Lead (Pb). Deterministic and stochastic approaches were explore to examine the human health risks. The evaluated Hazard Indices (HI) for the investigated mining locations are < 1, the recommended threshold provided by United State Environmental Protection Agency (USEPA) for acceptable non-cancer risk. The estimated cancer risk levels for the mining locations exceeds the acceptable range of 1.00E-6 and 1.00E-4.

• Thus, the mining is making significant contribution to HMs pollution, which is dangerous human health.
• However, the Monte Carlo simulation (MCS) reveals that the 95th, 50th and 5th percentiles of the cumulative probability of the cancer risks are within the acceptable range.
• This work will be useful for decision makers in mitigating heavy metals contamination due to mining activities.

## Graphical abstract

**Figure:**
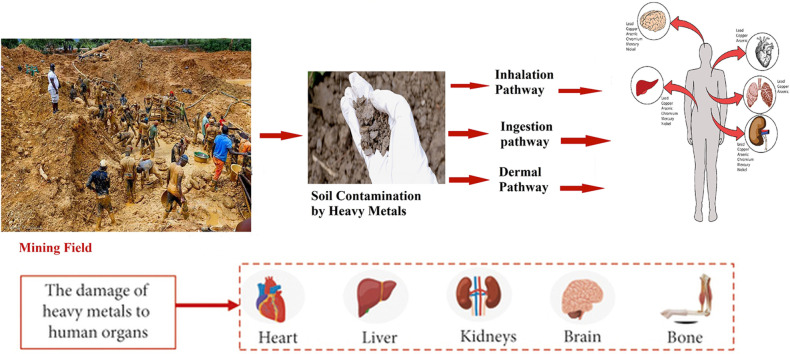

Specifications table

**Table:** 

Subject Area:	*Toxicity*
More specific subject area:	*Environmental Sciences*
Method name:	*Human Health Risk Assessment of Heavy Metals*
Name and reference of original method	*Monte Carlo approach to risks assessment of heavy metals.*
	*M.M. Orosun, A.D. Adewuyi, N.B. Salawu, M.O. Isinkaye, O.R. Orosun, and A.S. Oniku, Monte Carlo approach to risks assessment of heavy metals at automobile spare part and recycling market in Ilorin, Nigeria, Sci. Rep., 10 (2020) 22,084*. https://doi.org/10.1038/s41598–020–79,141–0.
Resource availability	*The data are available in this article.*

## Introduction

The continuous release of potentially toxic elements by anthropogenic activities like mining (particularly the type taking place at the gold and beryllium mining sites in Moro and Ifelodun, respectively) have been reported to bring about accumulation of the potentially toxic elements and other contaminants in the environment [1], [2], [3]. According to [1,[4], [5], [6], [7]] the presence of the PTEs in the human system is reported to bring about health effects manifesting differing symptoms, subject to the nature and amount of the chemical element ingested. While some potentially toxic elements are essential for human body at very small quantity, others like As, Pb, Cd, Cr and Ni are known human carcinogens [8]. When the presence of these toxic nuclides in the environment are enhanced, they will find their way into the human system either by ingestion, inhalation or dermal contact leading to severe health effects like cancer, damaging important body organs leading to death in some cases [4], [5], [6], [7], [8]. Tailings from the gold and beryllium mining activities are often dumped in the immediate environment in tons. This mining and dumping of tailings that usually contains higher concentrations of these poisonous elements is allegedly the leading cause of human exposure that could bring about severe human health effects like arsenicosis, cancer etc., damaging important body organs that could lead to death in some cases [4], [5], [6], [7], [8]. In spite of the massive mining activities that is going on in these areas, no study has been conducted on the environmental impacts of the mining activities until our earlier work [2], where onsite monitoring of activity concentrations of ^40^K, ^238^U and ^232^Th were carried out. This call for further investigation to reveal the extent of the potentially toxic elements contamination, since understanding of the origin of pollution, bioaccumulation, enrichment level, etc. are important contrivance in appraisal of the related health effects of the toxic elements on the inhabitants.

**Fig. 1:**
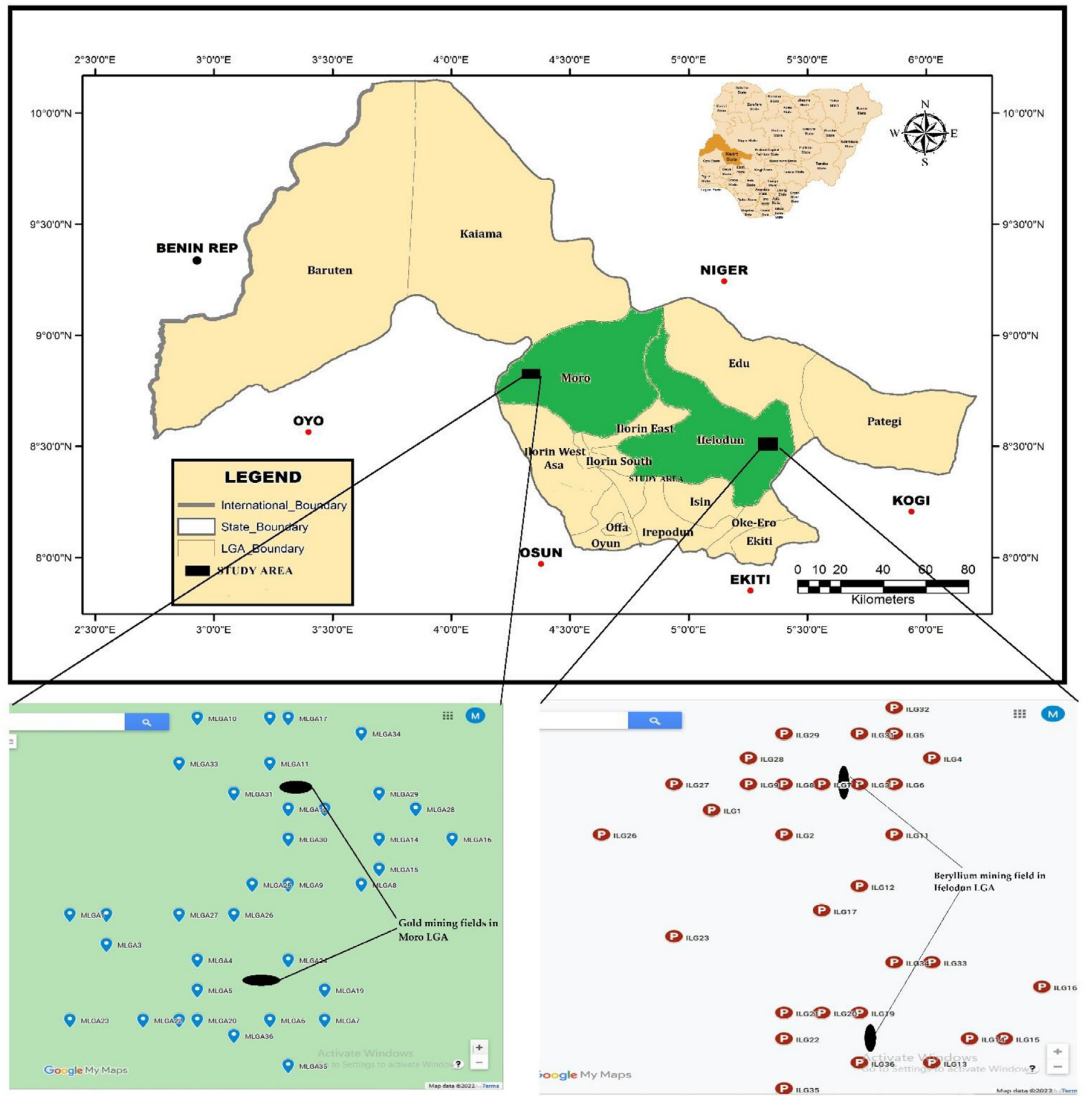
Map of the study area indicating the sampling points [2].

Consequently, the dataset from this study contains the mean value of the geochemical analyses of soil samples collected from beryllium and gold mining fields. The samples were filtered and analysed for the elements concentration using Atomic Absorption Spectrophotometer (AAS). The following Potentially Toxic Elements were detected from the analysed samples: Arsenic (As), Zinc (Zn), Cadmium (Cd), Nickel (Ni), Iron (Fe), Chromium (Cr), Manganese (Mn), Magnesium (Mg), Lead (Pb) and Copper (Cu). The result showing the statistical summary of the concentration of the potentially toxic elements in the mining fields as well as their corresponding means and geographical coordinates are presented in Tables 1 and 2. The statistical analysis was exploited to deepen our understanding on the geochemical relationship of the soil samples. In addition, this is done to enable comparison with the international recommended limit. The geological map of the study area is depicted in Fig. 1. Correlation analysis was conducted on the potentially toxic elements present in the soil samples and the result is as presented in Tables 3 and 4. Table 5 presents the results of the risk assessments.

**Table 1:** Descriptive Statistics of the measured heavy metals concentrations in the soil samples from the Gold mining field in Moro LGA.

Stat	Ni	Cu	Pb	Mn	Mg	As	Zn	Cd	Cr	Fe
Min	4.00	6.01	215.00	10.00	122.00	10.00	141.81	20.00	11.50	109.80
Max	10.01	58.01	643.01	21.00	252.00	30.00	260.00	92.24	91.20	152.80
Mean	6.37	24.61	448.75	15.86	174.56	23.17	202.63	46.37	62.45	127.58
St. Error	0.39	2.61	26.52	0.64	6.40	1.36	5.26	3.04	4.66	2.80
Median	5.26	19.28	456.03	16.48	170.08	28.40	200.35	43.06	74.15	120.58
SD	2.33	15.68	159.10	3.82	38.39	8.17	31.53	18.26	27.99	16.79
Variance	5.45	245.78	25,311.50	14.62	1473.57	66.69	994.19	333.40	783.19	281.76
Kurt	−1.42	−1.03	−1.52	−1.30	−0.72	−1.48	−0.84	−0.58	−1.16	−1.55
Skew	0.60	0.51	−0.22	−0.29	0.45	−0.69	0.27	0.39	−0.68	0.45
Range	6.01	52.00	428.01	11.00	130.00	20.00	118.19	72.23	79.70	43.00
Sum	229.23	886.02	16,155.01	570.92	6284.08	834.03	7294.81	1669.38	2248.08	4592.76
Count	36.00	36.00	36.00	36.00	36.00	36.00	36.00	36.00	36.00	36.00

**Table 2:** Descriptive Statistics of the measured heavy metals concentrations in the soil samples from the Beryllium mining field in Ifelodun LGA.

Stat	Ni	Cu	Pb	Mn	Mg	As	Zn	Cd	Cr	Fe
Min	6.00	2.00	6.01	6.01	72.01	5.01	128.00	12.00	6.80	21.10
Max	14.09	12.00	15.01	24.09	235.01	43.34	160.01	60.37	76.80	151.07
Mean	10.03	5.67	10.83	14.00	127.77	25.01	147.50	30.01	46.13	65.67
St. Error	0.40	0.47	0.47	0.92	6.45	1.54	1.83	1.88	4.76	4.78
Median	10.10	5.39	11.13	12.99	126.35	25.03	147.14	29.06	51.10	68.75
SD	2.43	2.83	2.85	5.53	38.69	9.27	10.96	11.30	28.58	28.70
Variance	5.89	8.03	8.10	30.56	1496.55	85.87	120.08	127.71	816.67	823.95
Kurt	−0.95	−0.39	−1.16	−0.95	0.69	−0.02	−1.06	0.66	−1.62	1.45
Skew	0.29	0.61	0.02	0.35	0.90	−0.20	−0.42	0.67	−0.38	0.55
Range	8.09	10.00	9.00	18.09	163.00	38.33	32.01	48.36	70.00	129.97
Sum	361.09	204.06	390.00	504.08	4599.69	900.38	5309.86	1080.23	1660.84	2364.26
Count	36.00	36.00	36.00	36.00	36.00	36.00	36.00	36.00	36.00	36.00

**Table 3:** Pearson's correlation matrix showing the relationship between the measured heavy metals from Moro Gold mining field.

	Fe	Ni	Cu	Pb	Mn	Mg	As	Zn	Cd	Cr
Fe	1.0000									
Ni	−0.0554	1.0000								
Cu	−0.0359	−0.1167	1.0000							
Pb	−0.1923	0.0059	−0.1180	1.0000						
Mn	−0.2480	−0.0582	0.2486	−0.0129	1.0000					
Mg	0.0359	−0.0663	−0.2301	−0.0062	−0.0473	1.0000				
As	−0.0229	0.0568	−0.0642	−0.1173	−0.0209	−0.0736	1.0000			
Zn	−0.3197	0.0754	−0.0886	0.3431	0.3612	−0.0315	0.1216	1.0000		
Cd	−0.2317	0.2025	−0.0330	0.0053	−0.1189	−0.1493	0.0693	−0.0111	1.0000	
Cr	0.1674	0.0007	−0.4266	−0.0608	−0.0580	0.2595	0.0846	−0.1785	0.0295	1.0000

**Table 4:** Pearson's correlation matrix showing the relationship between the measured heavy metals from Ifelodun Beryllium mining field.

	Fe	Ni	Cu	Pb	Mn	Mg	As	Zn	Cd	Cr
Fe	1.0000									
Ni	0.1390	1.0000								
Cu	0.0344	0.4326	1.0000							
Pb	−0.0715	−0.0006	0.0583	1.0000						
Mn	0.1732	0.1643	0.1358	0.0123	1.0000					
Mg	−0.1140	0.0224	−0.1942	0.0920	0.0043	1.0000				
As	0.0137	−0.0298	−0.0875	0.0086	−0.0245	−0.0831	1.0000			
Zn	−0.3457	0.0336	−0.0340	0.1035	0.0590	0.1037	−0.1162	1.0000		
Cd	0.1591	−0.0272	−0.1437	0.0067	0.2304	−0.0891	−0.0926	0.2619	1.0000	
Cr	−0.0918	0.0157	0.0668	0.1802	0.0395	0.1739	0.2781	0.0055	−0.0410	1.0000

**Table 5:** Mean CDI_ing_, CDI_inh_, CDI_derm_, THQ_ing_, THQ_inh_, THQ_derm_, HI and ILCR for both Moro and Beryllium mining fields.

LOCATIONS	CDI_ing_	CDI_inh_	CDI_derm_	THQ_ing_	THQ_inh_	THQ_derm_	HI	ILCR
Moro	1.62E-3	2.38E-7	1.03E-5	2.93E-1	9.08E-4	4.39E-2	3.38E-1	2.63E-3
Ifelodun	6.89E-4	1.01E-7	6.88E-6	8.32E-2	6.73E-4	3.97E-2	1.24E-1	1.73E-3

## Method details

The study focused on the measurement of the concentration of heavy metals herein referred to as potentially toxic elements (PTEs), in the samples of soil obtained from beryllium minefields and gold minefields in Ifelodun and Moro respectively, Kwara, Nigeria. The potentially toxic metals includes; Arsenic (As), Zinc (Zn), Cadmium (Cd), Nickel (Ni), Iron (Fe), Chromium (Cr), Manganese (Mn), Magnesium (Mg), Lead (Pb) and Copper (Cu). The soil samples were collected in duplicates using soil auger and packaged in a labelled polyethylene bags to avoid material reaction. The soil samples collected were digested by adding 3 ml of HNO_3_ and 9 ml of concentrated HCl. The solution was now heated on an electrical heating plate to boiling point for approximately 15 min. The digested solution was further filtered into standard flasks and then diluted with few ml of distilled water. The digested samples were measured into a plastic reagent bottle for Atomic Absorption Spectrophotometry. Mining and mineral processing activities is widely believed to be associated with introduction and enhancement of potentially toxic elements in the environment [1], [2], [3], [4], [5], [6], [7]. Amongst these toxic elements, Arsenic (As), Zinc (Zn), Cadmium (Cd), Nickel (Ni), Iron (Fe), Chromium (Cr), Manganese (Mn), Magnesium (Mg), Lead (Pb) and Copper (Cu) receives universal attention because their presence in the human system is reported to bring about health effects manifesting differing symptoms, subject to the nature and amount of the chemical element ingested [1,2]. When the presence of these toxic elements in the environment are enhanced by the mining activities, they will be introduced into the human system either by ingestion, inhalation or dermal contact [1], [2], [3], [4], [5], [6], [7]. Thus, data generated in this study could be used as baseline data for reference and future study of the level of potentially toxic elements contamination in soil of the mining regions. The data could be used for educational purposes, research and studies on environmental pollution to further broaden the information on potentially toxic element contamination in the environment. It could as well provide understandings on the effects of potentially toxic elements on the proximate inhabitants within the mining sites if the mean concentration level of the contaminants exceeds the standard regulatory limit of concentration and; give contextual appraisal for decision making on remediation and environmental protection policy in Kwara State and Nigeria.

### Mining locations

The study areas are located in Kwara State, Nigeria. The areas have been reported to be underlain by basement complex rocks, which are majorly represented by phyllites, gneisses, granodiorites, granites, schists and pegmatites [9,10]. Minerals ores are found to be linked with gneisses and schists in this part of the country [9]. Sandstone beds at the contact between the Cretaceous sediments of Nupe Basin and the Basement Complex in these areas are known to host gold, lead, beryllium among others [10]. Accordingly, the study area host beryllium and gold. Explicit geology of Kwara State have been reported [2,4,11].

### Sample collection and preparation

Seventy-two (72) soil samples were collected from the mining fields with thirty-six (36) samples each from Ifelodun beryllium mining field and Moro gold mining field. The samples were obtained in duplicates to generate the mean values and minimise error. The soil samples were collected using soil auger and were put in a black polyethylene bags of about 2 kg each and labelled for easy of identification.

The samples obtained from the locations went through a process of open room drying to obtain constant dry weight samples that are moisture free [11,12], pulverised and put through a sieve through a 1 mm sieve. One gram of the samples was placed in a digestion flask and three (3) ml of HNO_3_ that is concentrated and nine (9) ml of HCl was added [13]. The mixture was now heated on an electrical heating plate for fifteen (15) minutes until all brownish fumes were expelled out confirming the completion of the digestion of the samples. The digested solution was cooled and then filtered into a twenty-five (25) ml standard flask and then diluted with few ml of distilled water and then analysed using the Atomic Absorption Spectrophotometer [13].

The spectrometry analysis was carried out at ROTAS Soil-Lab Ibadan, Nigeria using Buck Scientific Atomic Absorption Spectrophotometer Model 210 VGP (Buck Scientific, E. Norwalk, CT, USA). Standard solutions (one-thousand (1000) ppm of each of the Potentially Toxic Elements) for calibration were procured from Inorganic ventures (300 Technology Drive, Christianburg, VA 24,073, USA), specifically prepared for Atomic Absorption Spectrophotometric Analysis. From each of these standards, stock solution of one-hundred (100) ppm was prepared using the dilution equation (Eq. (1)),

(1) $$C_{1}V_{1}=C_{2}V_{2}$$

where C_1_ is the known concentration of standard solutions one-thousand (1000) ppm, V_1_ is the unknown volume, C_2_ is the known concentration to be prepared one-hundred (100) ppm and V_2_ is the volume to be prepared one-hundred (100) ml. That is, C_1_V_1_ are the initial concentration and volume, and C_2_V_2_ are the final concentration and volume, respectively. Working standard solution was then prepared using the same formula from the stock solution in line with linear range of the metal to be analysed, which is in turn used to prepare the calibration curve. Blank samples were utilised to check and correct the reagents and distilled water background effects, and determine the limit of detection of the instrument. The limits of detection of the Atomic Absorption Spectrophotometer ranged from 0.005 ppm (Arsenic) to 0.080 ppm (Lead). Standard procedures were followed to ensure quality control. All the laboratory apparatuses were washed several times and rinsed with de-ionised water before each usage. Accuracy and precision of the procedures were ensued using the reagent blanks and the duplicate samples preparation [14].

### Descriptive statistics

The detailed statistical summary of the concentration of heavy metal analyzed in each mining field is presented in Tables 1 and 2. The estimated minimum, maximum, range, sum, mean, median, standard deviation, variance, Skewness and Kurtosis were shown in the tables. The measured values for all the heavy metals were slightly skewed (i.e. the distribution is approximately symmetric) since most of the measure of the asymmetry of their probability distribution about their means is in the range of −1 and +1 [14].

### Correlation analyses

The correlation analysis between the heavy metals measured was assessed using Pearson techniques (Tables 3 and 4). The results were categorised in accordance to the correlation coefficient R [2,15], as follows:

0.8 ≤ |R| ≤ 1 signifies a strong correlation;

0.5 ≤ |R| ≤ 0.8 signifies a significant correlation;

0.3 ≤ |R| ≤ 0.5 signifies a weak correlation; and

|R| < 0.3 signifies an insignificant correlation.

### Human health risk assessment (HHERA)

In this current research, the risk evaluation of the toxic elements ((Arsenic (As), Cadmium (Cd), Chromium (Cr), Nickel (Ni), Iron (Fe), Manganese (Mn), Magnesium (Mg), Zinc (Zn), Copper (Cu) and Lead (Pb)) was initiated by estimating the chronic daily intake (CDI) of each of the metals through the possible exposure pathways. The pathways in this case are; ingestion pathway (Eq. (2)), inhalation (Eq. (3)) and dermal contact (Eq. (4)) [11,12].

(2) $$CDI_{ing-soil}=\frac{Cs\times IngRs\times EF\times ED}{BW\times AT}$$

(3) $$CDI_{inh-soil}=\frac{Cs\times InhRs\times EF\times ED}{PEF\times BW\times AT}$$

(4) $$CDI_{derm}=\frac{C\times SA\times AF\times ABS\times EF\times ED}{BW\times AT}$$

where CDI_ing-soil_ is the chronic daily intake of the individual HMs in the ingested soil. Cs is the concentration of the HMs in the soils. BW is body weight of the exposed individual, ED is the lifetime exposure duration (year), IngRs is the ingestion rate of the contaminated soils, EF is the exposure frequency (day/year), and AT is time period over which the dose is averaged (day). PEF is the particle emission factor (m^3^/kg). SA is the exposed skin surface area (cm^2^), KP is the permeability constant of the skin, ABS is the skin absorption factor.

The Carcinogenic and Non-Carcinogenic Risk Assessment were calculated using the incremental lifetime cancer risk (ILCR**) (**Eq. (5)) [11,12] and the hazard index (HI) (Eq. (7)) [15].

(5) ILCR=CDI×SF

(6) $$HQ=\frac{CDI}{RfD}$$

(7) HI=∑HQ

where CDI is the chronic daily intake of a given toxic constituent and RfD is the reference dose for the element. SF (mg/L/day)^−1^ is the carcinogenic gradient factor.

### Monte Carlo simulation (MCS) using Oracle Crystal Ball

Monte Carlo simulation is a mathematical technique that is used to evaluate the probable outcomes of any event with uncertainties. This technique is the most widely used approach that accommodates the uncertainties, ambiguities, and variability linked with many risk-related problems particularly the ones that affect human safety and the ecosystem [4]. The daily rate of ingestion of the toxic substance (i.e. Cd, Cr, As, and Pb) by individuals that are exposed, the human body weight (assumed to be 70 kg in this study), the concentration of toxic metals in the samples collected from the mining sites, and the carcinogenic slope factor of the pollutants, are all sources of uncertainties. These uncertainties makes the evaluation of human health risk assessment somewhat complicated. Thus, estimation of the health risks values using the risk assessment model outlined in Section 2.5 of this work, either overestimates or underestimates the actual radiological risk. Consequently, a probabilistic approach using Monte Carlo simulation (MCS) that has the advantage of minimizing uncertainty was employed to inspect the probable cancer risks. Highlighted below is the stepwise procedure for the Monte Carlo simulation.

i. The first step of the MCS is to define hypotheses and give the probability distribution types and parameters of the input variables of the model [16,17]. The uncertain variables in this study as stated earlier include; daily rate of ingestion of the toxic substance; the human body weight; the amount of toxic metals in the samples; and the carcinogenic slope factors.
ii. The second step was to define the prediction variables according to the equation for the cancer risk, and then define the number of iterations (10,000 in this case) and start the simulation.
iii. The final step is to interpret the uncertainty of the predicted variables according to the operation results. The Monte Carlo Simulations was performed using the Oracle Crystal Ball software version 11.1.2.4.850.

Since HQ > 1 reveals an increased probability of unfavorable health effects to the exposed populace and HQ < 1 subsequently indicates no possibility of negative health effects [11,12,14], the risk of non-carcinogenic effects is less for both locations. Similarly, since cancer risk higher than $1\;\times 10^{-4}$ are considered high and values below $1\;\times 10^{-6}$ are assumed not to cause any cancer risk [11,12,14], it follows that the cancer risk is high for both lactations.

## Author contribution

Muyiwa Michael OROSUN conceived, designed the research work, performed the Monte Carlo Simulations and wrote the first draft of the paper. Muyiwa Michael OROSUN, Samuel O. INUYOMI, Hitler LOUIS, and Emmanuel Olusegun EHINLAFA collected the data, performed the risks analysis, and compilation of the work. Mojisola Rachael USIKALU, Hussein K. OKORO, Maxwell OMEJE, and Kayode John OYEWUMI ensured the supervision and final editing of the manuscript.

## Declaration of Competing Interest

The authors declare that they have no known competing financial interests or personal relationships which have or could have or could be perceived to have influenced the work reported in this article.

## Data Availability

Data will be made available on request.
